# A Putative Role of Vasopressin/Oxytocin-Type Neuropeptide in Osmoregulation and Feeding Inhibition of *Apostichopus japonicus*

**DOI:** 10.3390/ijms241814358

**Published:** 2023-09-20

**Authors:** Xiao Cong, Huachen Liu, Yingqiu Zheng, Muyan Chen

**Affiliations:** The Key Laboratory of Mariculture, Ministry of Education, Ocean University of China, Qingdao 266003, China; congxiao@stu.ouc.edu.cn (X.C.); liuhuachen@stu.ouc.edu.cn (H.L.); zhengyingqiu@stu.ouc.edu.cn (Y.Z.)

**Keywords:** vasopressin/oxytocin-type (VP/OT)-type neuropeptides, sea cucumber, echinoderm, contraction effects, osmoregulation, feeding inhibition

## Abstract

Vasopressin/oxytocin (VP/OT)-type neuropeptide is an ancient neurophysin-associated neuropeptide and has been intensively studied to be involved in multiple physiological processes in protostomian and deuterostome vertebrates. However, little is known about the functions of VP/OT-type neuropeptide in deuterostome invertebrates especially in echinoderms. Here, we firstly report VP/OT-type neuropeptide signaling in an important economic species, *Apostichopus japonicus*, which is widely cultured in Asia, with high nutritional and medicinal values. Molecular characterization analysis of holotocin and its precursor revealed the highly conserved features of VP/OT family. The candidate receptor for holotocin (AjHOR) was confirmed to be able to activate the signaling via cAMP-PKA and possible Ca^2+^-PKC pathway, and further activated the downstream ERK1/2 cascade. Holotocin precursor expression profile showed that they were mainly concentrated in circumoral nerve ring. Furthermore, in vitro pharmacological experiments demonstrated that holotocin caused contractile responses in preparations from *A. japonicus*. And in vivo functional studies indicated that short-term injection of holotocin resulted in body bloat and long-term injection resulted in reduced body mass, suggesting potential roles of holotocin in osmoregulation and feeding co-inhibition with holotocin–CCK. Our findings provided a comprehensive description of AjHOR–holotocin signaling, revealed ancient roles of holotocin in osmoregulation and feeding inhibition by controlling muscle contractions.

## 1. Introduction

Vasopressin/oxytocin (VP/OT) neuropeptide family is an ancient lineage [1] and one of the most intensively studied families [2]. Analysis of the phylogenetic distribution of neuropeptide signaling in the animal kingdom indicated that the evolutionary origin of VP/OT-type signaling can be traced to its common ancestor in Bilateria [3,4,5]. VP/OT neuropeptide was firstly characterized as a substance extracted from pituitary gland that could increase blood pressure in dogs [6], two structurally related neurohypophyseal hormone, VP and OT, were subsequently identified by purification and bioactivity detection of pituitary extraction [7]. The molecular features of VP (CYFQNCPRG-NH_2_) and OT (CYIQNCPLG-NH_2_) are extremely conserved throughout the Bilateria, including amino acid sequences, cyclic structure and C-terminal amidation [8], which indicated a common evolutionary origin of VP- and OT-type neuropeptides. It should be noted that VP/OT-type neuropeptides are now mainly named with “-tocin” as a suffix. In the jawless fish *Lethenteron japonicum*, there was one precursor gene encoding a VP/OT-type neuropeptide [9], and in gnathostomes and higher vertebrates, tandem duplication of the single VP/OT gene from a common ancestral gene of the gnathostomes was completed to produce two precursor genes encoding the VP and OT genes, respectively [9,10,11]. Furthermore, in almost all invertebrates, only a single member of VP/OT-type neuropeptide (VP or OT) family was identified [12,13,14,15].

As for VP/OT-type receptors, four types of receptors were initially isolated in mammals, three (V1a, V1b and V2) of them mediate the effects of VP, and the remaining one interacted with oxytocin [16,17,18,19]. Two rounds of whole-genome duplications (2R WGD) early in vertebrate evolution were well supported because four-way paralogous regions covered a large part of the human genome [20,21]. Therefore, Ocampo et al. (2012) [22] speculated that the four receptors mentioned above might also reflect 2R WGD. Sequence-based phylogenies indicated that two genes encoding VP/OT-type receptors had expanded to eight genes during the two rounds of whole-genome duplication in the early vertebrate evolution [22,23]. Nevertheless, diverse receptors of VP/OT-type neuropeptide were possessed in different vertebrates because of lineage-specific gene loss and additional gene/genome duplication [22,23,24]. VP/OT-type receptors were also reported in invertebrates. For example, one VP/OT-type receptor was identified in *Tribolium casteneum* [15], *Lasius niger* [25], *Cancer borealis* [26], *Eisenia foetida* [27], *Ciona intestinalis* [14], *Saccoglossus kowalevskii* [4] and *Asterias rubens* [2] and two receptors were identified in *Caenorhabditis elegans* [28] and *Platynereis dumerilii* [29,30]. In addition, there were three VP/OT-type receptors in *Octopus vulgaris* mediating two ligands [31,32].

Based on the genes/transcripts encoding VP/OT-type neuropeptide signaling identified from protostomian and deuterostomian animals, the physiological effects of VP/OT superfamily signaling were analyzed and compared [33,34,35,36]. In protostomes, multiple physiological functions of VP/OT-type neuropeptides in different Phylum have been characterized. Vasopressin-like peptide in arthropod *T. casteneum* had a diuretic activity [37], and “inotocin” signaling in *Lasius neglectus* was of vital importance in energy regulation [38]. “Nematocin” in nematode *C. elegans* demonstrated a neuro-modulatory role in associative learning [28] and also in reproductive behavior by both central and peripheral actions [39]. In vitro pharmacological research indicated that “sepiatocin” could cause tonic contraction on the oviduct, penis and vena cava from molluscs *Sepia officinalis* [40], and “Lys-conopressin” in *Lymnaea stagnalis* regulated reproductive physiology [41,42]. And “annetocin” in annelid *E. foetida* induced pulsatile contractions in quiescent nephridia and potentiated spontaneous rhythmic contractions or pulsatile contractions on other preparations [13].

In deuterostomes, initial investigation of physiological roles indicated that VP increased blood pressure and had antidiuretic activity, and OT caused the uterine contraction and stimulated lactation in mammals [7]. The conservative functions of VP/OT-type neuropeptide in vertebrates have been extensively studied, mainly including water homeostasis, reproduction and social behavior [43,44,45]. However, few functional studies of VP/OT-type neuropeptide in deuterostomian invertebrates especially in echinoderms have been reported. Related physiological studies of echinoderm VP/OT-type neuropeptide were only characterized in sea urchin (*Echinus esculentus*), starfish (*A. rubens*) and feather star (*Antedon mediterranea*). “Echinotocin” identified in *Strongylocentrotus purpuratus* caused dose-dependent contraction in the preparations from the sea urchin *E. esculentus*, including esophagus and tube feet [46]. Recent research has reported that “crinotocin” in *A. mediterranea* induced arm contractions and the function of “crinotocin” was mediated by excitable cells [47]. On the contrary, “asterotocin” in *A. rubens* acted as a muscle relaxant to induce the cardiac stomach eversion [2]. However, nothing is known about the effects of VP/OT-type neuropeptide in Holothuroidea species like *Apostichopus japonicus*.

Sea cucumber *A. japonicus*, as a deuterostomian invertebrate, occupied an “intermediate” phylogenetic position relative to the vertebrates and protostomian invertebrates [48] and has a great ecological restoration capability [49]. Furthermore, it is an essential economic Holothuroidea species in Asian aquaculture because of its significant nutritional and medicinal value [50,51]. *A. japonicus* has a large market demand, but there are bottlenecks for the development of its aquaculture. Feeding and osmoregulation are two of the most important physiological processes for survival, and they need to be optimized in non-natural culture environment for marine animals like sea cucumbers. So far, relative studies for these two important physiological processes in *A. japonicus* focused on the basic physiological and biochemical indexes [52,53,54] and potential regulatory genes [55,56] and lacked the exploration of the impact of neuropeptide regulations. Neuropeptides, synthesized and secreted by neuronal cells, have been reported to regulate various important behavioral and physiological processes and have also promoted the development of aquaculture technology in sea cucumbers [57,58]. Our previous study has constructed the most complete neuropeptide database for *A. japonicus* [59] including holotocin. However, so far only kisspeptin-type [60,61], L-type SALMFamide [62], luqin-type [63] and pedal peptide-type [64] neuropeptides have been investigated functionally. 

Based on that, our present study compared the molecular characterization of the VP/OT-type signaling system in different species, analyzed the expression profile of holotocin precursor in *A. japonicus*, validated the candidate receptor, confirmed the signaling system and investigated the in vitro and in vivo functions of holotocin in *A. japonicus*. This is the first study to investigate the potential physiological roles of VP/OT-type neuropeptides in echinoderm Holothuroidea that will provide new insights for the evolution of VP/OT-type signaling and enrich the functional research on echinoderm neuropeptides. 

## 2. Results

### 2.1. Molecular Characterization of the VP/OT-Type Signaling System in the Sea Cucumber A. japonicus

**Figure 1:**
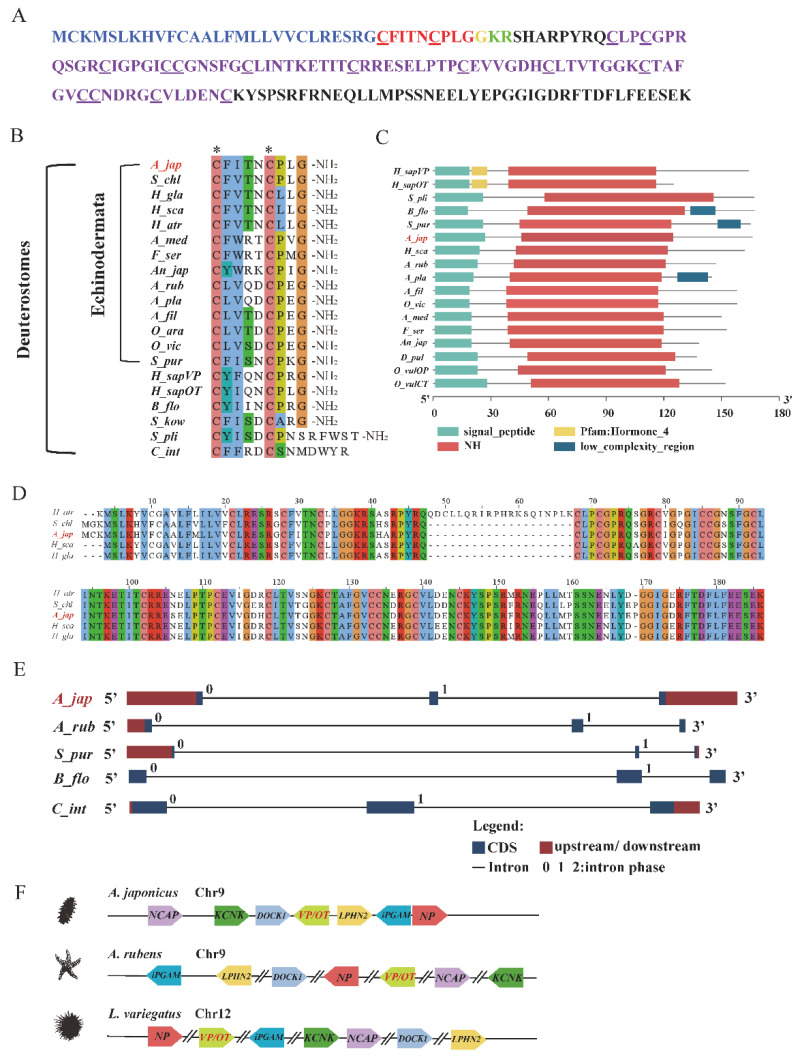
Characterization of holotocin, holotocin precursor and holotocin precursor genes in *A. japonicus.* (**A**) Amino acid sequences of *A. japonicus* holotocin precursor with the predicted signal peptide are shown in blue, a predicted cleavage site is shown in green, holotocin is shown in red, a C-terminal glycine that is a putative substrate for amidation is shown in orange and the neurophysin domain is shown in purple. The two red underlined cysteines form disulfide bridges, and the cysteines in neurophysin domain are shown with a purple underline. (**B**) Comparison of VP/OT-type neuropeptides from deuterostomes. The conserved pair of cysteine residues was shown by *. The GenBank numbers are shown in the Supplementary Table S1. (**C**) Conserved domains of protein sequences from VP/OT family. Gray line represent the length of proteins, and domains are represented by rectangles. (**D**) Comparison of VP/OT-type neuropeptide precursor proteins in Holothuroidea. The same amino acids are colored. (**E**) DNA structure of VP/OT-type neuropeptide precursor genes. Exons are represented by blue rectangles, introns are represented by lines and upstream and downstream are represented by red rectangles. The position of intron phase is represented by numbers. (**F**) Synteny of *A. rubens*, *L. variegatus* and *A. japonicus* VP/OT-type neuropeptide precursor genes. The chromosome location of VP/OT-type neuropeptide precursor genes is designated, and the relative orientation of adjacent genes is shown and various genes are marked in different colors. Transcriptional direction of genes is shown by arrows. *NCAP*, condensin complex subunit; *KCNK*, potassium channel subfamily K; *DOCK1*, dedicator of cytokinesis protein 1; *LPHN2*, latrophilin-2; *iPGAM*, 2,3-bisphosphoglycerate-independent phosphoglycerate mutase; and *NP*, nucleolar protein.

The full-length cDNA of holotocin precursor of *A. japonicus* contained a 501 bp ORF (open reading frame) encoding 163 amino acids (Supplementary Figure S1A) comprising one predicted signal peptide region in N-terminal, one VP/OT-type neuropeptide, one cleavage site (KR) and one neurophysin domain with fourteen cysteines (Figure 1A). Homology comparison revealed that except in squirt (*Styela plicata* and *C. intestinalis*), VP/OT-type neuropeptides including holotocin in deuterostomes have conserved features like disulfide bond between the first and the sixth cysteines and the C-terminal glycine amide (Figure 1B, Supplementary Figure S1B). Furthermore, VP/OT-type neuropeptides in Holothuroidea (*A. japonicus*, *Holothuria glaberrima*, *Holothuria scabra*, *Holothuria atra* and *Stichopus chloronotus*), shared more common characterizations, and they had a Cys-Phe-X-Thr-Asn-Cys-X-Leu-Gly (CFXTNCXLG) motif (in which X is variable). More interestingly, *H. glaberrima*, *H. scabra* and *H. atra* belonging to Holothuria had the same VP/OT-type neuropeptides (CFVTNCLLG) and their position 7 was occupied by a leucine residue (Leu; L), while in Stichopus (*A. japonicus and S. chloronotus*), it was a typical proline residue (Pro; P) at that position. Furthermore, VP/OT-type neuropeptides in Asteroidea (*A. rubens*, *Acanthaster planci*) and Ophiuroidea (*Ophiopsila aranea*, *Amphiura filiformis*) also shared the same VP/OT-type neuropeptides, respectively. (Figure 1B). The protein structure of holotocin predicted using SWISS-MODEL is shown in Supplementary Figure S1C. Furthermore, domain prediction of protein sequences from VP/OT family indicated that holotocin presented the signal peptide and neurohypophysial hormones’ (NH) domains (Figure 1C). Homology analysis of VP/OT-type neuropeptide precursors in Holothuroidea (*A. japonicus, H. atra, S. chloronotus, H. glaberrima* and *H. scabra*) showed that 69% amino acids of these proteins were identical (Figure 1D). The exon/intron structure analysis of the holotocin precursor gene of *A. japonicus* was determined from the genome, as well as those in *A. rubens*, *S. purpuratus*, *Branchiostoma floridae* and *C. intestinalis*, and they all showed three exons interrupted by two introns. And VP/OT-type precursors from these species all had a phase 0 intron interrupted exons encoding the N-terminal part of the precursors followed by a phase 1 intron interrupting exons encoding the C-terminal part of the precursors (Figure 1D). 

Synteny analysis was performed to investigate the orthology relationships of VP/OT-type precursor genes in echinoderms, and the neighboring gene environment of VP/OT genes was determined in *A. japonicus* and compared to those in *A. rubens* and *Lytechinus variegatus*. As shown in Figure 1E, the VP/OT-type precursor of *A. japonicus* located on chromosome 9 and was surrounded by *NCAP*, *KCNK*, *DOCK1*, *LPHN2*, *iPGAM* and *NP.* In *A. rubens* and *L. variegatus*, the VP/OT-type precursor was located on chromosome 9 and chromosome 12, respectively, and also positioned in genomic regions containing common neighboring genes of *A. japonicus*.

The candidate receptor sequence of holotocin cloned from *A. japonicus* contained a 1290 bp ORF encoding 429 amino acids with 35 potential phosphorylation sites (Supplementary Figure S2A). Transmembrane structure analysis showed that the candidate receptors of holotocin (AjHOR) includes seven transmembrane domains, which is a typical feature of GPCR [65], and the transmembrane positions are listed in Supplementary Figure S2B. Furthermore, we predicted the tertiary structure of AjHOR, and it is shown in Supplementary Figure S2C. To examine the relationship of AjHOR with VP/OT-type receptors from other species, the phylogenetic tree was constructed by choosing NPS/CCAP-type/NG peptide receptors as the paralogs of VP/OT-type receptors [66], and TRH-type receptors was selected as an outgroup. The results revealed that AjHOR is the ortholog of VP/OT-type receptors. Specifically, AjHOR was clustered with the predicted starfish (*A. rubens* and *Patiria miniata*) and sea urchin (*S. purpuratus* and *L. variegatus*) VP/OT-type receptors (Supplementary Figure S3).

### 2.2. Analysis of Holotocin Precursor Expression Profile and AjHOR Relative Transcript Expression Levels

**Figure 2:**
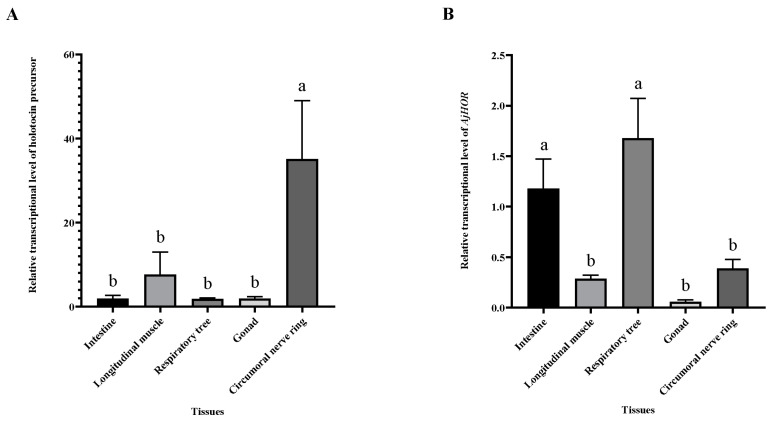
Holotocin precursor and *AjHOR* relative transcript expression levels in different tissues of *A. japonicus*. (**A**) Holotocin precursor gene and (**B**) *AjHOR*. The significant difference (*p* < 0.05) is shown by different lowercase letters. Values are mean ± S.E.M (n = 5 biological replicates).

**Figure 3:**
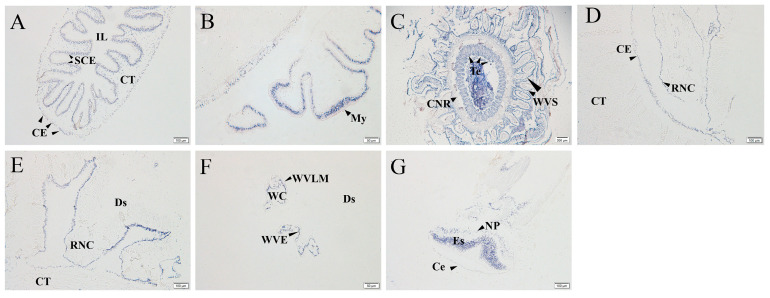
Localization of holotocin precursor in *A. japonicus* using mRNA in situ hybridization. (**A**) Intestine. (**B**) Mesentery. (**C**) Oral position: water vascular system, tentacle and circumoral nerve ring. (**D**) Longitudinal muscle. (**E**) Body wall. (**F**) Water canal. (**G**) Tube feet. Ce, cuticle; CE, coelomic epithelium; CNR, circumoral nerve ring; CT, connective tissue; Ds, dermis; Es, epidermis; IL, intestine lumen; My, mesentery; NP, nerve plexus; RNC, radial nerve cord; SCE, simple columnar epithelium; Te, tentacle; WC, water canal; WVS, water vascular system; WVE, water vascular epidermis; and WVLM, water vascular longitudinal muscle. Holotocin precursor signals in different tissues were shown by arrows. Scale bar: 200 µm in (**C**); 100 µm in (**A**,**D**,**E**,**G**); 50 µm in (**B**,**F**).

The expression levels of holotocin precursor in different tissues were investigated using qRT-PCR (real-Time quantitative PCR). It was found that expression levels of holotocin precursor in the circumoral nerve ring was significantly higher than those in the intestine, longitudinal muscle, respiratory tree and gonad (*p* < 0.05) (Figure 2A). *AjHOR* was dominant in the respiratory tree and intestine and was also expressed in other tissues including longitudinal muscle (Figure 2B). In addition to quantitative analysis, holotocin precursor expression pattern in different tissues was further investigated using in situ hybridization. The results revealed a widespread expression pattern of holotocin precursor in different tissues (Figure 3), and negative control (holotocin precursor sense probe) is shown in Supplementary Figure S4.

In intestine, holotocin precursor signals were observed in connective tissue and coelomic epithelium, and most stained cells were concentrated on the simple columnar epithelium (Figure 3A). Furthermore, positive signals on mesentery surrounding the intestine were also present (Figure 3B). The sea cucumber nervous system comprises radial nerve cords that extend along the longitudinal muscles and linked by a circumoral nerve ring located in the peristome [67]. The oral position of sea cucumber *A. japonicus* mentioned here included the water vascular system, tentacles and circumoral nerve ring, and all of which showed the extensive and intense staining patterns for holotocin precursor (Figure 3C). In addition to the circumoral nerve ring, positive signals were also observed in the radial nerve cord of the nervous system (Figure 3D,E). In longitudinal muscle, holotocin precursor signals were detected in coelomic epithelium and connective tissue (Figure 3D). In body wall, positive stains were observed in dermis and in the water vascular epidermis and longitudinal muscle of water canal (Figure 3E,F). From the outside to the luminal side, the sucking disc of the tube feet of *A. japonicus* consists of cuticle, epidermis, nerve plexus, connective tissue, end plate and mesothelium. The longitudinal muscular layer attached to both sides of the end plate [68]. Holotocin precursor positive signals were also observed in the tube feet including the cuticle, epidermis and nerve plexus regions (Figure 3G).

### 2.3. AjHOR Is Activated by Holotocin

**Figure 4:**
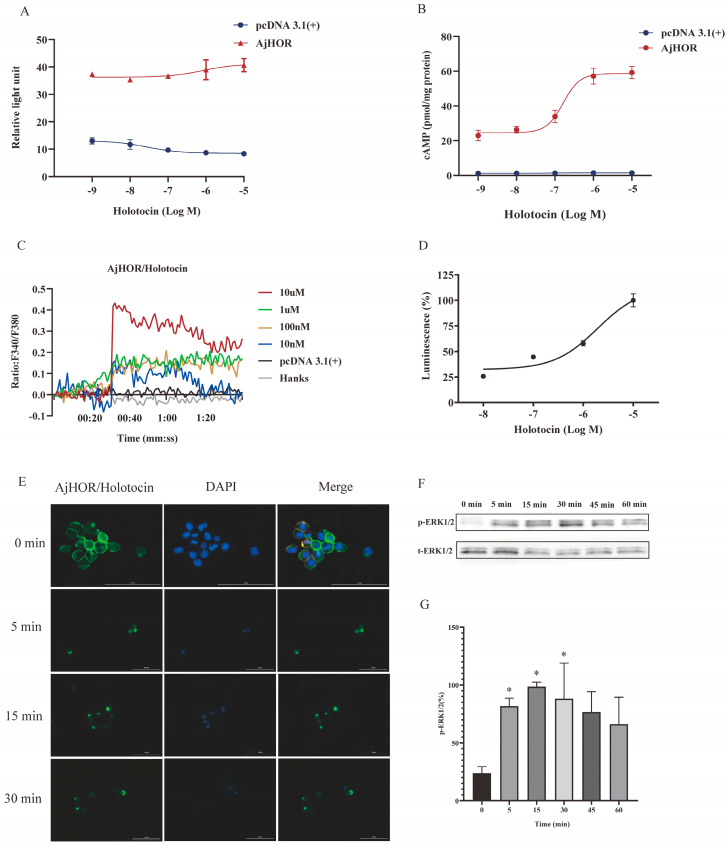
Functional characteristics of holotocin signaling system. (**A**) CRE-driven luciferase activities in *AjHOR*-expressing HEK293T cells was detected. (**B**) Quantitative detection of cAMP accumulation in *AjHOR*-expressing cells under the stimulation of holotocin (10^−9^–10^−5^ M). Intracellular Ca^2+^ mobilization in *AjHOR*-expressing HEK293T cells was measured in response to holotocin (10^−8^–10^−5^ M) (**C**), and holotocin caused concentration-dependent activation of *AjHOR* (**D**). (**E**) Internalization of *AjHOR*/pEGFP-N1 was initiated by 10^−6^ M holotocin in *AjHOR*/pEGFP-N1-expressing HEK293T cells after incubation with indicated times. Cell nucleus were stained by cell nucleus probe (DAPI). Scale bar: 100 µm. (**F**) Immunoblot intensity of representative bands. Holotocin stimulated phosphorylation of ERK1/2 in *AjHOR*-expressing HEK293T cells, which were incubated with indicated times. (**G**) The normalized p-ERK1/2 according to t-ERK1/2. Error bars represent SEM for three independent experiments. All pictures and data are representative for at least three independent experiments. * Significant differences between 0 min and treated groups with *p* < 0.05.

To examine whether or not AjHOR is activated by holotocin, the CRE (cAMP—response element)-Luc (Luciferase) reporter genes assay was performed to indirectly detect the intracellular cAMP level. As shown in Figure 4A, holotocin obviously increased in co-transfected cells, and cells transfected with the pcDNA3.1(+) exhibited no response upon the stimulation of holotocin. To further test the effect of holotocin, we detected the intracellular accumulation of cAMP directly using ELISA analysis, and the data indicated that holotocin significantly elicited a concentration-dependent increase in the cAMP level with EC_50_ = 9.408 × 10^−7^ M (Figure 4B). Furthermore, holotocin also triggered intracellular Ca^2+^ mobilization with EC_50_ = 1.949 × 10^−6^ M (Figure 4C,D), and AjHOR-induced intracellular Ca^2+^ mobilization was found to be sensitive to EGTA (Supplementary Figure S5). Based on ligand binding and activation, GPCRs internalization from the cell surface to the cytoplasm was performed and thereby G protein-dependent signaling was attenuated [69,70]. Fusion expression of *AjHOR*/pEGFP-N1 was used to visualize the process of internalization in HEK293T cells, and internalization was observed since HEK293T cells with *AjHOR*/pEGFP-N1 were stimulated by 10^−6^ M holotocin for 5 min (Figure 4E). These results revealed that AjHOR was activated by holotocin, suggesting the presence of a functional VP/OT-type neuropeptide signaling in *A. japonicus*.

Activated GPCRs’ signaling to the mitogen-activated protein kinase (MAPK) cascades was considered as an important functional outcome of GPCR activation [71]. Therefore, we investigated whether holotocin could induce intracellular ERK1/2 phosphorylation through AjHOR. As shown in Figure 4F,G, stimulation with holotocin caused the activation of AjHOR, inducing significant ERK1/2 activation with maximal phosphorylation at 15 min and dephosphorylation at 45 min. These results provided the evidence that holotocin could active AjHOR and further activated the downstream ERK1/2 cascade.

### 2.4. Holotocin Causes Dose-Dependent Contractions in Intestine, Longitudinal Muscle and Tentacle Preparations from A. japonicus

**Figure 5:**
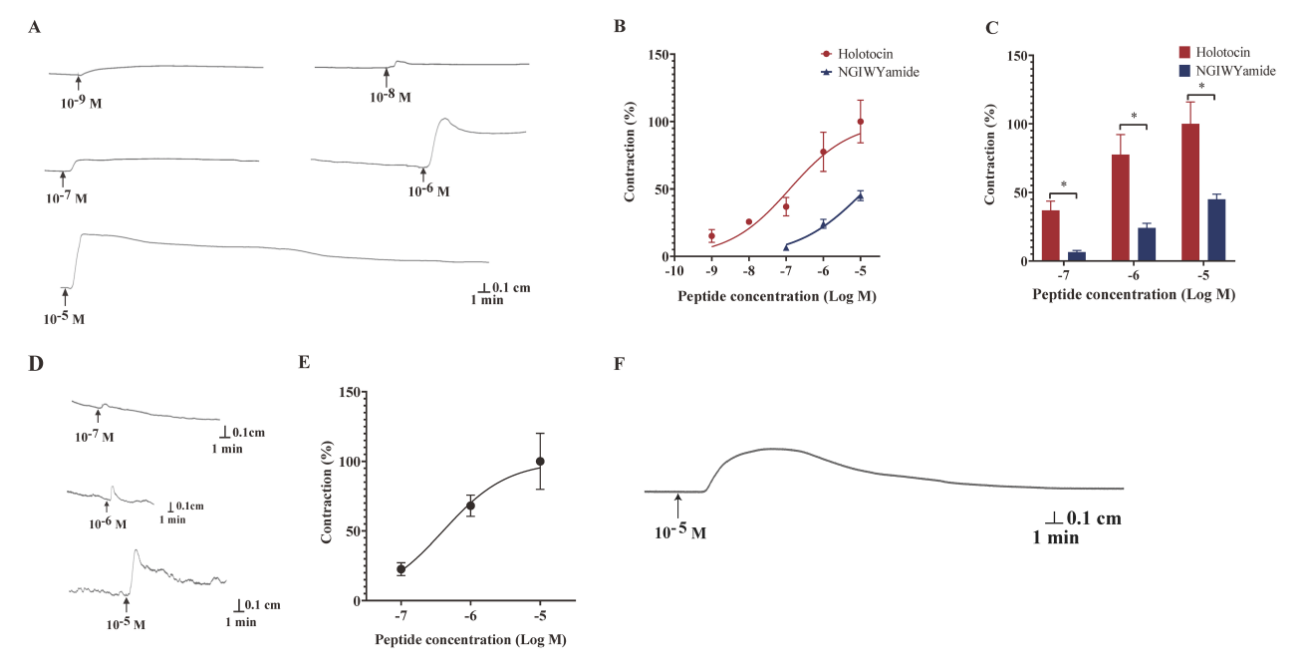
Holotocin caused contractions in longitudinal muscle, tentacle and intestine from *A. japonicus*. (**A**) A representative recording showed that holotocin (10^−9^–10^−5^ M) caused tonic contraction of longitudinal muscle preparation. (**B**) Graph showed the dose-dependent contracting effect of holotocin (10^−9^–10^−5^ M) and NGIWYamide (10^−7^–10^−5^ M) on longitudinal muscle preparations. (**C**) Comparison of contraction efficiency of holotocin and NGIWYamide at different concentrations, and the contraction efficiency of holotocin in 10^−5^ M was defined as 100%. (**D**) A representative recording showed that holotocin (10^−7^–10^−5^ M) caused tonic contraction of intestine preparation. (**E**) Graph showed the dose-dependent contracting effect of holotocin on intestine preparations. Responses were expressed as the mean percentage contraction (mean ± S.E.M.; n = 3). (**F**) The effect of holotocin (10^−5^ M) on tentacle preparation in vitro. * The effect of muscle contractions induced by holotocin and NGIWYamide at the same concentration was significantly different (*p* < 0.05).

Previous research has reported that the VP/OT-type neuropeptide has both relaxant and contractile effects in echinoderms (*E. esculentus*, *A. rubens*) [2,46]. To investigate the function of holotocin, longitudinal muscle, intestine and tentacle from *A. japonicus* were prepared. For longitudinal muscle preparations, we observed that holotocin caused tonic and dose-dependent contraction at concentrations of 10^−9^–10^−5^ M, and the most obvious contraction effect was observed at 10^−5^ M (Figure 5A–C). More interestingly, the contraction effect at a concentration of 10^−5^ M could persist for at least 15 min (Figure 5A). NGIWYamide neuropeptide (10^−7^–10^−5^ M) reported to cause contraction of longitudinal muscle preparations from *A. japonicus* [67] was used to compare the efficacy of holotocin, and the contraction forces of NGIWYamide are shown in Supplementary Figure S6A. By comparing the tonic contraction effect of NGIWYamide and holotocin, we found that the mean magnitude of the contracting action of holotocin was 5.7 times, 3.2 times and 2.2 times higher (a significant increase) than those of NGIWYamide at concentrations of 10^−7^, 10^−6^, and 10^−5^ M, respectively (Figure 5B,C) (*p* < 0.05). Holotocin caused dose-dependent and tonic contraction in intestine preparations from *A. japonicus* at concentrations between 10^−7^ and 10^−5^ M (Figure 5D,E). The most obvious contraction was observed at 10^−5^ M holotocin (Figure 5E). Furthermore, 10^−5^ M holotocin also induced contraction of tentacle (Figure 5F, Supplementary Figure S6B). The negative control is shown in Supplementary Figure S6C. 

Furthermore, Ca^2+^-deprivation experiments were conducted to investigate the source of calcium ions during holotocin-induced longitudinal muscle contraction. The results revealed that 10^−6^ M holotocin caused longitudinal muscle contraction in artificial sea water with 0 mM and 10 mM Ca^2+^, but had no effect on the longitudinal muscle in the Ca^2+^-free solution of chelating agent (Supplementary Figure S7A). Furthermore, the effect of holotocin in 0 mM Ca^2+^ artificial sea water and Ca^2+^-free solution of chelating agent was 28.4% and 10.2% of the effect in 10 mM Ca^2+^ artificial sea water and showed significant difference (*p* < 0.05) (Supplementary Figure S7B).

### 2.5. Holotocin-Induced Osmoregulation and Feeding Inhibition In Vivo

**Figure 6:**
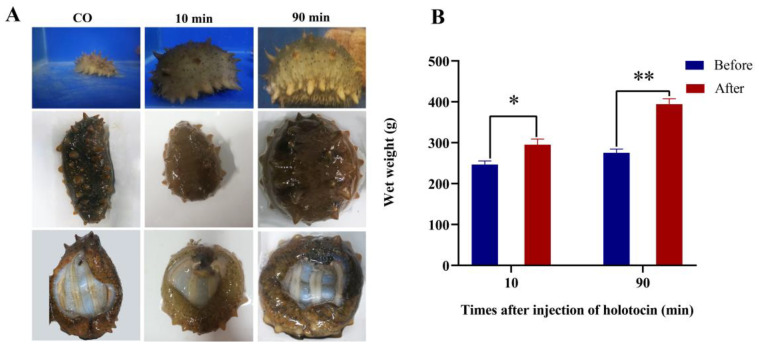
Physiological response of holotocin in *A. japonicus*. (**A**) Morphological changes in *A. japonicus* after injection with seawater or 10^−5^ M holotocin. (**B**) Comparison of wet weight before and after 10 min or 90 min of holotocin injection. The significant difference (*p* < 0.05) is shown by *, and *p* < 0.01 is shown by **.

Informed by the in vitro research, we further assessed the impact of holotocin in vivo in *A. japonicus*. Based on the in vitro experiment, 10^−5^ M holotocin was selected to inject into the abdomen of *A. japonicus*. After 10 min and 90 min of injection, we observed that the body shape of sea cucumbers became bloated in comparison with the control group (Figure 6A), and the mean wet weight gained was 48.7 g and 118.9 g, respectively, which was significantly different compared with the control group (*p* < 0.05) (Figure 6B). 

**Figure 7:**
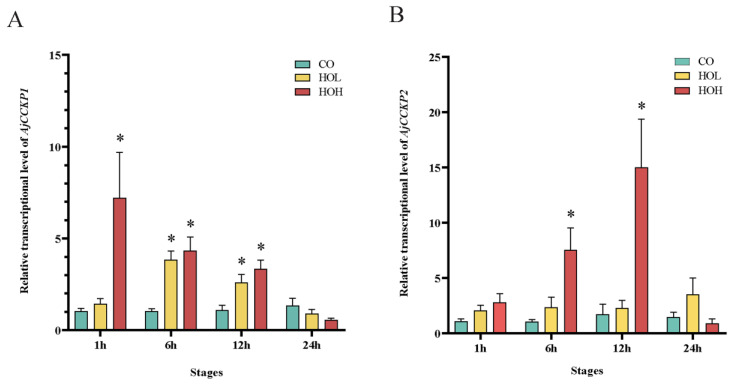
*AjCCKP1* and *AjCCKP2* relative transcript expression levels in different stages after injection of holotocin in *A. japonicus*. (**A**) *AjCCKP1* was tested after injection of holotocin with indicated times. (**B**) *AjCCKP2* was tested after injection of holotocin with indicated times. The significant difference (*p* < 0.05) is shown by *. Values are mean ± S.E.M. (n = 5 biological replicates). CO: control group (sterilized seawater); HOL: holotocin low concentration group (5 × 10^−3^ mg/mL); and HOH: holotocin high concentration group (5 × 10^−1^ mg/mL).

It has been reported that VP/OT-type signaling is involved in the feeding regulation [72,73]. In the present study, we also investigated the effects of holotocin on food intake in *A. japonicus,* and the expression levels of two cholecystokinin precursors (*AjCCKP1* and *AjCCKP2*) were tested. As indicated in Figure 7, *AjCCKP2* expression level in intestine was only significantly up-regulated after 6 h and 12 h of injection with HOH (*p* < 0.05), and *AjCCKP1* was significantly up-regulated after 6 h and 12 h of injection with HOL and after 1 h, 6 h and 12 h of injection with HOH (*p* < 0.05). Considering that in the Bilateria, CCK was a typical feeding inhibitory regulator [74], we preliminarily concluded that holotocin might have interacted with AjCCK1/AjCCK2 and could be involved in feeding inhibition.

**Figure 8:**
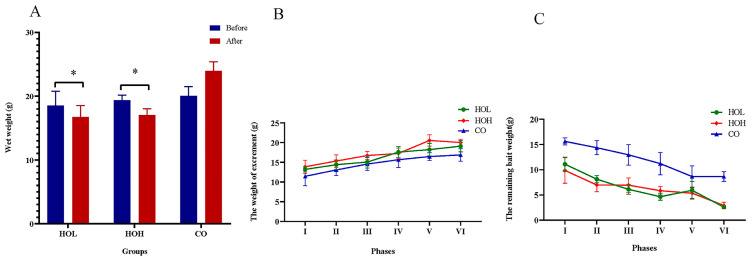
Effect of holotocin injection on growth of *A. japonicus*. (**A**) Wet weight changes before and after injection of holotocin in different groups. The significant difference (*p* < 0.05) is shown by *. (**B**) Calculation of the residual bait weight in different phases. (**C**) The feces weight in different phases after injection of holotocin in different phases. CO: control group (sterilized seawater); HOL: holotocin low concentration group (5 × 10^−3^ mg/mL); and HOH: holotocin high concentration group (5 × 10^−1^ mg/mL).

To further verify the inhibitory effect of holotocin on feeding, a long-term injection experiment was carried out. The wet weights of sea cucumbers injected with HOL or HOH were significantly decreased after 24 days injection (*p* < 0.05), and no significant difference was detected in CO (Figure 8A). In HOL and HOH groups, the remaining bait weight gradually increased with phase progression, and accordingly, the weight of excrement gradually decreased (Figure 8B). The intestinal anatomy status for different treatments are shown in Supplementary Figure S8. The experimental results provided strong evidence that holotocin could inhibit feeding.

## 3. Discussion

Accumulating studies have reported physiological functions of VP/OT-type neuropeptides in vertebrates [8]. However, although it is a popular and important neuropeptide, studies in invertebrates especially in echinoderms are rarely reported. The present study firstly focused on an economically important echinoderm, the sea cucumber *A. japonicus,* to explore the expression pattern and functional characteristics of VP/OT-type neuropeptides. 

It has been reported that only a single VP/OT-type neuropeptide exists in almost all invertebrates [75]. In the present study, we also confirmed a VP/OT-type neuropeptide holotocin in *A. japonicus* with a conserved disulfide bond and an amidation modification at the C-terminal, and these features are comparable with the conservative characteristics of vertebrates and protostomian invertebrates [8], suggesting that the evolutionarily conserved protein structure of VP/OT-type neuropeptide can be traced back to a common ancestor in Bilateria. Furthermore, holotocin precursor of *A. japonicus* has a 14 cysteines-containing neurophysin domain in C-terminal, which was consistent with the finding that the link between neurophysins and VP/OT-type neuropeptides was evolutionarily conserved in Bilateria [14,15,41,76]. This domain has been reported to be essential for the packaging, processing and protection of the neurohypophyseal hormones VP and OT, and it is required by them to properly target and regulate secretory pathways [77]. Noteworthily, homology analysis further showed the highly conserved features of VP/OT-type neuropeptides and corresponding precursors in Holothuroidea, especially in Holothuria, and they shared the same VP/OT-type neuropeptides. Combining the sequence analysis from other echinoderms like Asteroidea and Ophiuroidea, we speculated that VP/OT-type neuropeptides were highly conserved in each class of Echinodermata. Domain analysis of VP/OT neuropeptide precursors from different species revealed that holotocin had a highly conserved signal peptide and NH domains in bilaterians. 

Exon/intron structure analysis revealed that three protein-coding exons and a phase 0 and a phase 1 intron were all present in analyzed VP/OT-type precursor genes from deuterostomes, which is consistent with previous studies, indicating that the VP/OT-type precursors have three exons and another evolutionarily conserved feature is the presence, location and phase of introns [78,79]. Therefore, the conserved structural organization of genes here provided the strong evidence of VP/OT-type precursor genes orthology in deuterostomes. Synteny analysis indicated that the transcriptional direction and the position of genes relative to the VP/OT-type precursors were quite different in echinoderms, which is inconsistent with the findings in five selected bony vertebrate species (human, western clawed frog, chicken, spotted gar and medaka) [80], suggesting lineage-specific evolution in echinoderms. 

Cumulative data have revealed that VP/OT-type neuropeptide has effects on reproduction (*C. elegans*, *C. intestinalis*), muscle relaxation and feeding (*A. rubens*), muscle contraction (*S. plicata*, *O. vulgaris*) and water/salt homeostasis (*T. casteneum*) [36,37,39,76,81,82]. In our present study, analysis of holotocin precursor and holotocin expression profiles also showed an extensive expression pattern that was predominantly distributed in the circumoral nerve ring, which suggested that the VP/OT-type neuropeptide in sea cucumber also performs complicated and diverse roles in multiple physiological processes.

To further investigate holotocin signaling, the candidate receptor of holotocin was predicted and validated. AjHOR has been reported to be a member of the rhodopsin-like GPCRs belonging to the GPCR A family [65]. Present phylogenetic analysis also revealed that AjHOR was clustered with VP/OT-type receptors from protostomes and other echinoderms, demonstrating that the evolutionary origin of VP/OT-type receptor can at least be traced to a common ancestor in all species of Bilateria.

CRE-Luc activity and intracellular cAMP level detection further confirmed the ligand-receptor relationship between holotocin and AjHOR, which then triggered the AjHOR signaling via the cAMP-PKA pathway. Furthermore, holotocin-induced concentration-dependent intracellular increase in calcium was also detected in the present study. Correspondingly, both activation pathways of VP/OT-type neuropeptide, reported here, were also reported in *C. elegans* [28], suggesting that they are the classical signaling pathways of VP/OT-type neuropeptide in Bilateria. However, whether Ca^2+^ signaling pathway was activated by classical Ca^2+^-PKC pathway still needs to be further verified. AjHOR was also identified as a holotocin receptor by the internalization results. Furthermore, downstream ERK1/2 cascade activation were realized. All of above findings in present study suggested that holotocin mediated the conjugation of the receptor and G protein and then activated downstream signaling pathways via cAMP/PKA/MAPK or possible Ca^2+^/PKC/MAPK cascade to regulate physiological processes. This is the first study to identify the AjHOR–holotocin signaling in *A. japonicus* and makes it possible to further explain physiological function of this signaling system. 

**Figure 9:**
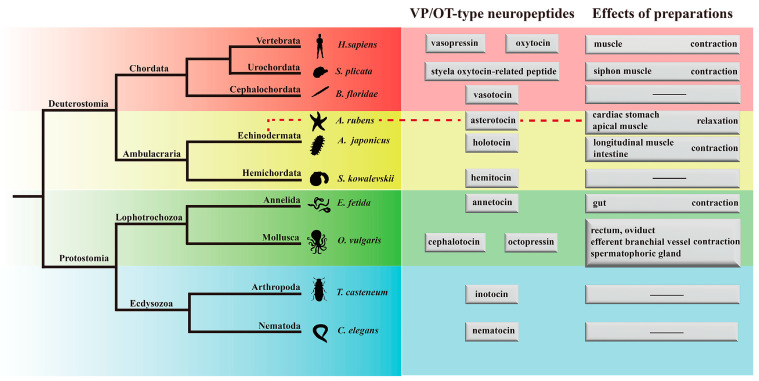
Diagram showing VP/OT-type neuropeptides from representative species in the Bilateria and the corresponding pharmacological effects in vitro. Species are classified by different colors, a dash (−) indicates that VP/OT-type neuropeptides were not functionally validated in vitro. Because of the specificity of asterotocin function, the diagram summarizes the functions of echinoderms by presenting the function of *A. rubens* in addition to summarizing the functions of *A. japonicus* in this study.

Consistent with the holotocin precursor expression pattern, in vitro pharmacological experiments indicated that holotocin caused contractile responses in longitudinal muscle, tentacle and intestine of *A. japonicus*, and this contractile effect of tissues is consistent with the myoexcitatory function of VP/OT-type neuropeptides in vertebrates and protostomian invertebrates, like *O. vulgaris* and *S. plicata* [76,81]. All these studies suggested that the contractile effect of VP/OT-type neuropeptide is evolutionarily conserved. Contractile effects of neuropeptides on the intestine and tentacles suggested its potential function in feeding. In addition, the contractile effects of holotocin and NGIWYamide on longitudinal muscles was compared, and we found that holotocin is much more effective and potent than NGIWYamide in *A. japonicus*. Interestingly, holotocin had a sustained tonic contractile effect on longitudinal muscle lasting as long as 15 min, and this phenomenon was not observed for other neuropeptides identified in *A. japonicus* [67,83], suggesting that holotocin is by far the most effective neuropeptide for causing contractile effect on longitudinal muscle in *A. japonicus*. Noteworthily, asterotocin acts as a muscle relaxant in *A. rubens* [2], and as illustrated in Figure 9, the relaxing effect of VP/OT-type neuropeptide was not found in other studied species. Therefore, these findings demonstrated the muscle activated function of VP/OT-type neuropeptide were diverse in echinoderms. In addition, Ca^2+^-deprivation experiments and EGTA pretreated cell calcium measurement experiment indicated that the process of holotocin-induced longitudinal muscle contraction requires extracellular calcium ions.

In vivo actions of holotocin were also investigated, and the body bloat of *A. japonicus* was observed by injecting 10^−5^ M holotocin and that further supported that holotocin can cause tonic longitudinal muscle contraction in vivo and in vitro.

Previous studies had indicated that coelomic fluid of echinoderms is isosmotic to the environment [84,85]. Coelomic fluid volume adjustment is one of the most effective ways for regulating osmotic pressure in *A. japonicus* [86], which may be attributed to muscle-induced water absorption, a specific regulatory mechanism for coelomic fluid and further leads to body bloat. Accordingly, the osmotic pressure that we tested here showed no significant difference, and this is probably due to the fact that the coelom of *A. japonicus* is an open system and the osmoregulation is an instantaneous process. Therefore, we further speculated that the body bloat of *A. japonicus* might be related to the regulation of osmotic pressure by holotocin, and this effect is fast. It has been reported that the VP/OT-type neuropeptide is involved in tonic contraction of siphon muscles in *S. plicata* and has a VP-like role in osmoregulatory processes [76]. And it is also well known that VP-type neuropeptides could conduct the osmoregulation in vertebrates [8,43,44]. This accumulating evidence suggested that VP/OT-type neuropeptides including holotocin had a potential evolutionary and ancient role in osmoregulation. After injection of holotocin, intestines in HOH and HOL were significantly thinner than those in CO, and weights of feces were significantly decreased, and the residual bait weights were gradually increased, suggesting a decreased intake in *A. japonicus*. Correspondingly, wet weights of sea cucumber in HOL and HOH were also significantly decreased. Therefore, all evidence demonstrated that holotocin had an inhibitory effect on feeding. In present study, we selected CCK, a typical feeding inhibitory regulator in the Bilateria [74], to confirm the inhibitory effect on feeding was the co-inhibition results of holotocin and CCK, indicating that the function of holotocin was similar to the typical role of OT in mammals [87,88,89,90]. Furthermore, holotocin–CCK induced co-inhibition is also supported by Ho et al. (2013) [91]. So far, the feeding-related physiological function of VP/OT-type neuropeptides in other deuterostomian invertebrates has also been studied, and accumulating evidence has suggested that VP/OT-type neuropeptides are potential ancient food intake regulators in Bilateria. For example, asterotocin was found to be a potent relaxant of cardiac stomach and triggered extra-oral feeding behavior in *A. rubens* [2]. Furthermore, the contractile effect of echinotocin on the sea urchin oesophagus [46] and in protostomes, the tonic contraction of octopressin in the *O.vulgaris* rectum [81] and enhancement of spontaneous rhythmic contractions of annetocin in *E. foetida* gut [13] also supported that VP/OT-type neuropeptides may be involved in the regulation of feeding-related processes. Notably, both holotocin precursor and *AjHOR* were expressed in the intestine and longitudinal muscle, suggesting that holotocin could directly exert biological activities on these tissues to regulate osmotic pressure and inhibit feeding in *A. japonicus.*

## 4. Materials and Methods

### 4.1. Animals

Adult sea cucumbers (*A. japonicus*) were obtained from an aquaculture farm in Weihai, Shandong, China. Animals were maintained in a seawater aquarium at about 18 °C and were fed on commercial formulated diet once a day for at least one week before they were sacrificed. Different tissues (intestine, longitudinal muscle, respiratory tree, gonad and circumoral nerve ring) were dissected from five adult sea cucumbers and kept at −80 °C for the following RACE and qRT-PCR studies. In histological studies, intestine, mesentery, water vascular system, circumoral nerve ring, tentacle, longitudinal muscle, body wall and tube feet preparations from three adult sea cucumbers were dissected and fixed in 4% paraformaldehyde (PFA) for in situ hybridization experiment. For pharmacological experiments, thirty-six sea cucumbers (~120 g body weight) were used for in vitro functional analysis, thirty sea cucumbers (~230 g body weight) and one hundred and fifty sea cucumbers (~20 g body weight) were used for in vivo functional validation.

### 4.2. Sequences’ Cloning and Confirmation and Analysis of Holotocin Precursorand AjHOR Sequences

The holotocin precursor sequence of *A. japonicus* (GenBank accession number: MF401997.2) has been predicted in our previous study [59]. For identifying candidate receptors of holotocin–AjHOR, the sea star *A. rubens* VP/OT-type receptor sequence (GenBank accession number: MK279533.1) [2] was submitted as a query to do a local BLASTn analysis based on *A. japonicus* circumoral nerve ring transcriptome database (GenBank: GHCH00000000.1) [59], and the e-value was set to 1. Informed by the published sequence of holotocin precursor and the obtained sequence of *AjHOR* (GenBank accession number: PIK60987.1) by local BLASTn analysis, Primer3Plus (https://www.primer3plus.com/, accessed on 28 March 2023) was used to design the gene-specific primers (Supplementary Table S2) for confirming the full-length of holotocin precursor and *AjHOR*. SMARTer RACE 5′/3′ Kit (Takara, Cat No. 634858) was applied according to the manufacturer’s instructions. The specific primers designed above and Universal Primer Mix (UPM) were used for the touchdown PCR, and the target products were then purified using the FastPure Gel DNA Extraction Mini Kit (Vazyme, Cat No. DC301). The concentration of cDNA was measured with NanoDrop 2000 (Thermo, USA), and its integrity was monitored using 1% agarose gels before the purified cDNA was cloned into the linearized pRACE vector (Takara, Cat. No. 639648). Then, the accuracy of the sequences was confirmed by sequencing (Beijing Genomics institution, China). Exon/intron structure of genes encoding the holotocin precursor gene was analyzed and compared with the VP/OT-type neuropeptide precursors from other species using the online tools Splign (https://www.ncbi.nlm.nih.gov/sutils/splign/splign.cgi, accessed on 3 May 2023) and GSDS2.0 (http://gsds.gao-lab.org/, accessed on 3 May 2023). And IBS 1.0 was used to present synteny of VP/OT-type neuropeptide genes. Open Reading Frame Finder (https://www.ncbi.nlm.nih.gov/orffinder/, accessed on 8 July 2023) was used to find the corresponding open reading frame (ORF) of genes, and the corresponding coding amino acid sequences of the genes were analyzed using sequence translation tool (http://www.bio-soft.net/sms/index.html, accessed on 8 July 2023). In order to find the conserved characteristics of VP/OT-type neuropeptides from *A. japonicus* and other species, Clustal-X was used to align the sequence with default settings and then edited using Jalview. Furthermore, online tool MEME was also used to identify the conserved motifs. To compare the sequences of VP/OT-type neuropeptide precursor proteins in Holothuroidea, Jalview was used and the conservation value was set to 100%. The Batch SMART plugin of TB tools (Toolbox for biologists) was used to compare and predict the conserved domains of protein sequences from VP/OT family. Tertiary structure of proteins was predicted with SWISS-MODEL (https://swissmodel.expasy.org/interactive, accessed on 8 July 2023). 

For the candidate receptor of holotocin, the procedure involving the ORF region and the corresponding amino acids sequences of the gene and the tertiary structure of protein was also performed as described above. Furthermore, the seven transmembrane domains were predicted using Protter 1.0 (http://wlab.ethz.ch/protter/start/, accessed on 1 August 2023), and NetPhos-3.1 (https://services.healthtech.dtu.dk/service.php?NetPhos-3.1, accessed on 1 August 2023) was used to predict the phosphorylation site. To construct Neighbor-Joining (NJ) phylogenetic tree of VP/OT-type receptors, MEGA 7.0 was used with default settings and then modified using iTOL (https://itol.embl.de/login.cgi, accessed on 1 August 2023). The detailed information for all sequences used for analysis are listed in Supplementary Tables S1 and S3.

### 4.3. Quantification Analysis of Holotocin Precursor/AjHOR and Localization of Holotocin Precursor in A. japonicus

Total RNAs were isolated using Trizol RNA isolation reagent (Vazyme, Cat No. R401-01) according to the instructions. A total of 500 ng RNA was used to synthesize cDNA by Hifair^®^ Ⅲ 1st Strand cDNA Synthesis SuperMix (YEASEN, Cat No. 11141ES60), and the expression levels were detected using Hieff UNICON Universal Blue qPCR SYBR Green Master Mix (YEASEN, Cat No. 11184ES08) with a Corbett Rotoe-Gnen Q (Qiagen, Germany). The *β*-actin and *β*-tubulin were selected as housekeeping genes according to the previous study [92]. The specific primers for holotocin precursor and *AjHOR* qRT-PCR analysis are listed in Supplementary Table S2. Relative expression levels were analyzed using the 2^−ΔΔCT^ method. Statistical analysis was carried out using one-way analysis of variance (ANOVA), followed by a Tukey post hoc test (IBM SPSS 25.0 software, Chicago, IL, USA). The significance threshold was limited to *p* < 0.05 for all tests. For in situ hybridization of holotocin precursor, sense and antisense digoxigenin (DIG)-labeled riboprobes were synthesized according to the method described previously [63], and the specific primers are listed in Supplementary Table S2. The method employed for holotocin precursor localization in preparations from *A. japonicus* was the same as described by Li et al. (2022) [63].

### 4.4. Validation of Holotocin Candidate Receptor 

To verify whether holotocin was a ligand of the candidate VP/OT-type receptor predicted in present study, pcDNA 3.1(+) vector and pEGFP-N1 vector comprising a partial Kozak consensus sequence (AAA) [93] before the ATG and the complete ORF of candidate VP/OT-type receptor were constructed based on the synthetic holotocin. After transfection or co-transfection of constructed plasmids into HEK293T cells, CRE-Luc detection, cAMP accumulation assay, intracellular calcium measurement, receptor localization and translocation assay and Western blotting were performed. All these methods have been described previously [60,63,94]. In brief, for localization and translocation assay, HEK293T cells transfected with *AjHOR*/pEGFP-N1 were stimulated with holotocin dissolved in serum-free medium (10^−6^ M), and the result was visualized with Automatic Intelligent Imaging Analysis System (Agilent BioTek, America). For CRE-Luc detection, HEK293T cells co-transfected with *AjHOR*/pcDNA 3.1(+) and pCRE-Luc were incubated with holotocin (10^−9^–10^−5^ M) for 4 h at 37 °C. Luciferase activity was detected using a firefly luciferase reporter gene assay kit (MK, MF4001) following the manufacturer’s instructions. For cAMP accumulation, cells transfected with the *AjHOR*/pcDNA 3.1(+) were stimulated by holotocin (10^−9^–10^−5^ M) for 15 min after starvation for two hours, and then detected using the cAMP Assay kit (R&D systems, Cat No. KGE002B). For the intracellular calcium measurement assay, HEK293T cells transfected with *AjHOR*/pcDNA 3.1(+) were loaded with 3 μM Fura-2/AM and incubated at 37 °C for 30 min and finally stimulated by holotocin (10^−8^–10^−5^ M). Ca^2+^ levels were measured using Synergy H1 Hybrid Multi-Mode Reader (Biotek, America). When required, cells were pretreated for 1 h with 5 mM EGTA in Hanks before loading with Fura-2/AM. For Western blotting, HEK293T cells expressing AjHOR were starved for 2 h, and incubated for indicated times (0 min, 5 min, 15 min, 30 min, 45 min and 60 min) with holotocin (10^−6^ M). Proteins were obtained and probed with antibody against phosphorylated ERK1/2 kinases followed by probing with goat anti-rabbit horseradish peroxidase-conjugated secondary antibody (Absin, Abs20040ss). Finally, RVL-100-G (ECHO, America) was used to visualize the PVDF membrane.

### 4.5. In Vitro Pharmacological Analysis of Holotocin on Intestine, Tentacle and Longitudinal Muscle Preparations from A. japonicus

Holotocin (CFITNCPLG-NH_2_, disulfide bridge between the two cysteines, C-terminal amidation) was synthesized by GL Biochem (China). Synthetic holotocin was applied in vitro to investigate its effect on intestine, tentacle and longitudinal muscle preparations from *A. japonicus*, with the NGIWYamide neuropeptide as a positive control confirmed to cause contraction of longitudinal muscles [67]. Firstly, the longitudinal muscle and intestine of *A. japonicus* were dissected and cut into strips approximately 20 mm in length. Then, these preparations were put into a 50 mL glass organ bath containing 20 mL sterilized seawater at about 15 °C to maintain physiological status and then tied with cotton ligatures at both of their ends. One of the ligatures was attached to a fixed metal hook and the other ligature was tied to a High Grade Isotonic Transducer (ADinstruments MLT0015, Oxford, UK) connected with PowerLab data acquisition hardware (ADinstruments PowerLab 4/26, Oxford, UK). Output signal (cm) from Powerlab was calculated using LabChart (v8.0.7) software.

The preparations were allowed to stabilize in sterilized seawater for about 20 min with 1 g resting tension to reach a stable baseline state. Then, 20 μL holotocin dissolved in sterilized seawater was added into an organ bath to achieve the test concentration, and each concentration was tested using three preparations. The same volume of sterile seawater was used as the negative control. To further investigate the source of calcium ions in the process of holotocin-induced contraction of the longitudinal muscles, the Ca^2+^-deprivation experiment was carried out. In brief, sterilized seawater was substituted for artificial sea water with different calcium ion concentration (Ca^2+^-free solution containing 0 mM, 5 mM and 10 mM EGTA) as described by Suzuki et al. (1982) [95], and contraction intensity of longitudinal muscles was analyzed after being stimulated by 10^−6^ M holotocin. 

### 4.6. In Vivo Functional Analysis of Holotocin on A. japonicus

We further investigated the effect of holotocin in vivo in *A. japonicus.* Firstly, thirty sea cucumbers were starved for a day before the experiment to normalize their physiological status and divided into three groups (10 individuals per group) randomly. Holotocin dissolved in sterilized seawater was injected into the abdomen of the sea cucumbers by intraperitoneal injection with a concentration of 10^−5^ M (1 ul g^−1^ wet weight). The control group was injected with equal amount of sterilized seawater instead of holotocin. Before and after injecting (10 min or 90 min), the wet weights of sea cucumbers were measured, the changes in body shape of sea cucumbers were captured with a camera (Brinno TLC200, China), and sea cucumbers were dissected to compare muscle status in different groups. Experimental data were analyzed with IBM SPSS Statistics 25, and *p*-value < 0.05 was considered as a significant difference.

To investigate the effect of holotocin on food intake and growth in *A. japonicus,* 5 × 10^−3^ mg/mL and 5 × 10^−1^ mg/mL holotocin was obtained by dissolving in sterilized seawater. For short-term injection experiment, a total of sixty sea cucumbers were randomly divided into 12 groups, four groups of twenty sea cucumbers were control groups (CO) and were injected with sterilized seawater (1 μL/g wet weight), and the eight experimental groups were evenly divided into high concentration groups (5 × 10^−1^ mg/mL, HOH) and low concentration groups (5 × 10^−3^ mg/mL, HOL). Then, intestines from sea cucumbers in CO, HOL and HOH groups were collected at different time points (1, 6, 12 and 24 h) after injection. Previous study had reported that cholecystokinin (CCK) was the putative endogenous satiety peptide [96] and was involved in food intake regulation [97], and AjCCK1 and AjCCK2 were also identified in *A. japonicus* based on our previous study [59]. In present study, the expression levels of *AjCCKP1* and *AjCCKP2* (GenBank accession number: MH636358 and MH351773) were detected as described above to explore the relationship between AjCCK1/AjCCK2 and holotocin in food intake. 

For long-term injection experiment, a total of ninety sea cucumbers were applied. Thirty sea cucumbers were divided randomly for each group (HOH, HOL and CO) and injected with holotocin (5 × 10^−3^ mg/mL or 5 × 10^−1^ mg/mL; 1 μL/g wet weight) or sterilized seawater from peristome once in 2 days. Wet weights of them were measured before the experiments and after 24 days of holotocin treatment. The remaining bait and excrement were collected by filtered fabric and weighted after dried every day. The collected data were analyzed using IBM SPSS Statistics 25, and *p*-value < 0.05 was considered as a significant difference.

## 5. Conclusions

**Figure 10:**
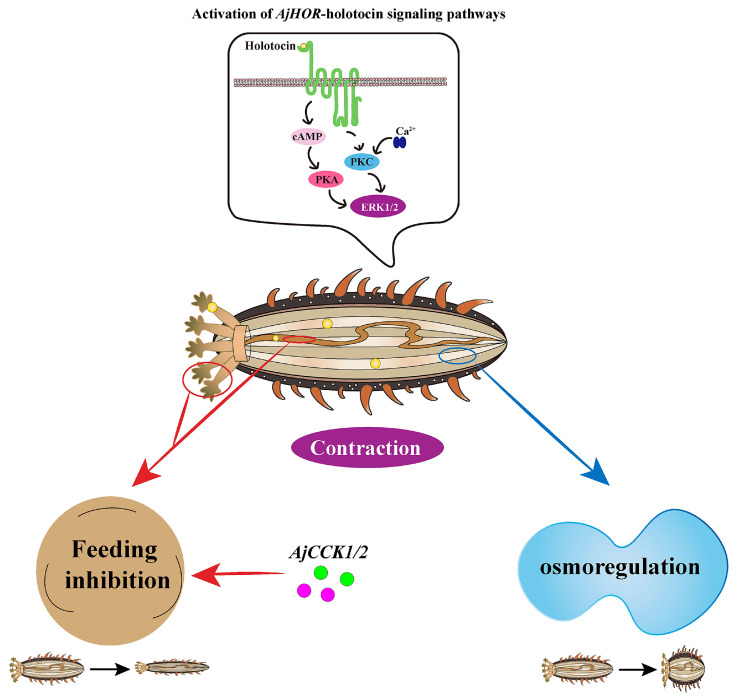
A schematic of holotocin’s potential physiological functions in *A. japonicus*. Holotocin are represented by the yellow circles, *AjHOR* are represented by green line. AjCCK1 and AjCCK2 are represented by pink and green circles respectively.

In summary, VP/OT-type signaling system in sea cucumber *A. japonicus* was characterized comprehensively. Holotocin, as a single member of VP/OT-type neuropeptides in *A. japonicus,* was also verified to have VP/OT-type neuropeptides conserved functions, including VP-type osmoregulation and OT-type feeding inhibition. For osmoregulation, we speculated that holotocin acted on longitudinal muscle to increase tension, thus causing the quick body bloat to regulate osmotic pressure, and this process was dependent on calcium ions. For feeding inhibition, we suggested that it was caused by holotocin-induced intestine and tentacle contraction, and may be co-inhibited by holotocin and CCK (Figure 10). However, further research is needed on how neuropeptides affect the expression of CCK and jointly regulate food intake. Taken together, our present findings supported the evolutionarily ancient role of VP/OT-type neuropeptides and provided new insights into osmoregulation and feeding regulation in marine invertebrates.

## Data Availability

Data are contained within the article.

## Supplementary Materials

The following supporting information can be downloaded at: https://www.mdpi.com/article/10.3390/ijms241814358/s1.
