# Supplementary material for: A Putative Role of Vasopressin/Oxytocin-Type Neuropeptide in Osmoregulation and Feeding Inhibition of Apostichopus japonicus
Source: Int J Mol Sci. 2023 Sep 20;24(18):14358. doi: 10.3390/ijms241814358 (PMC10532012; doi:10.3390/ijms241814358)
Supplement: Supplementary file 1 [file ijms-24-14358-s001.zip › Supplementary Figures.pdf]

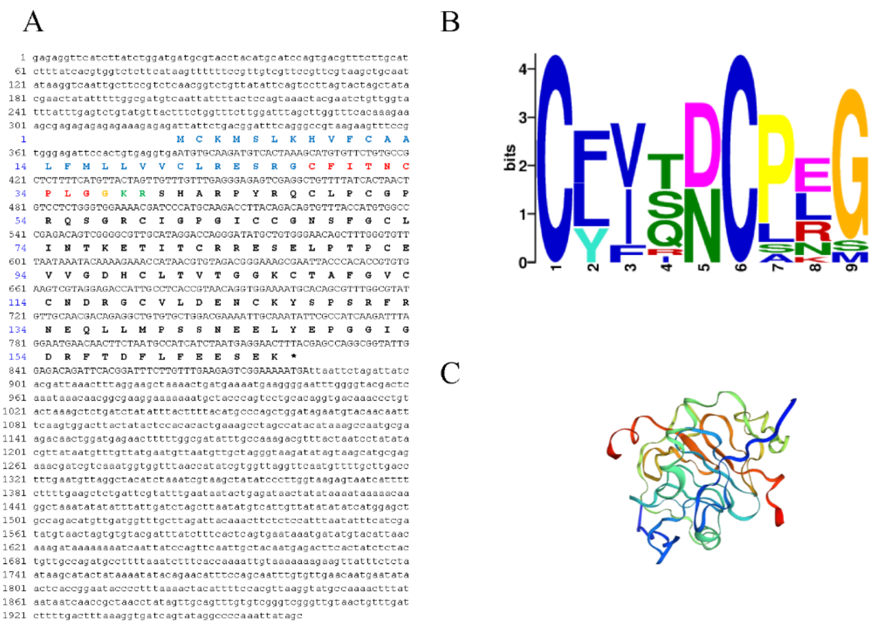

Figure S1. Holotocin in *A. japonicus* and the conserve sites of VP/OT-type peptides. (**A**) Nucleotide sequence of holotocin precursor(GenBank accession number: MF401997.2) and the deduced amino acid sequences. ORF was showed in uppercase; 3’UTR and 5’UTR were shown in lowercase. Predicted signal peptide was shown in blue, a predicted cleavage site was shown in green, holotocin was shown in red and a C-terminal glycine which was a putative substrate for amidation was shown in orange. (**B**) MEME was used for predicting conserve sites of VP/OT-type peptides. (**C**) The protein tertiary structure of holotocin precursor.

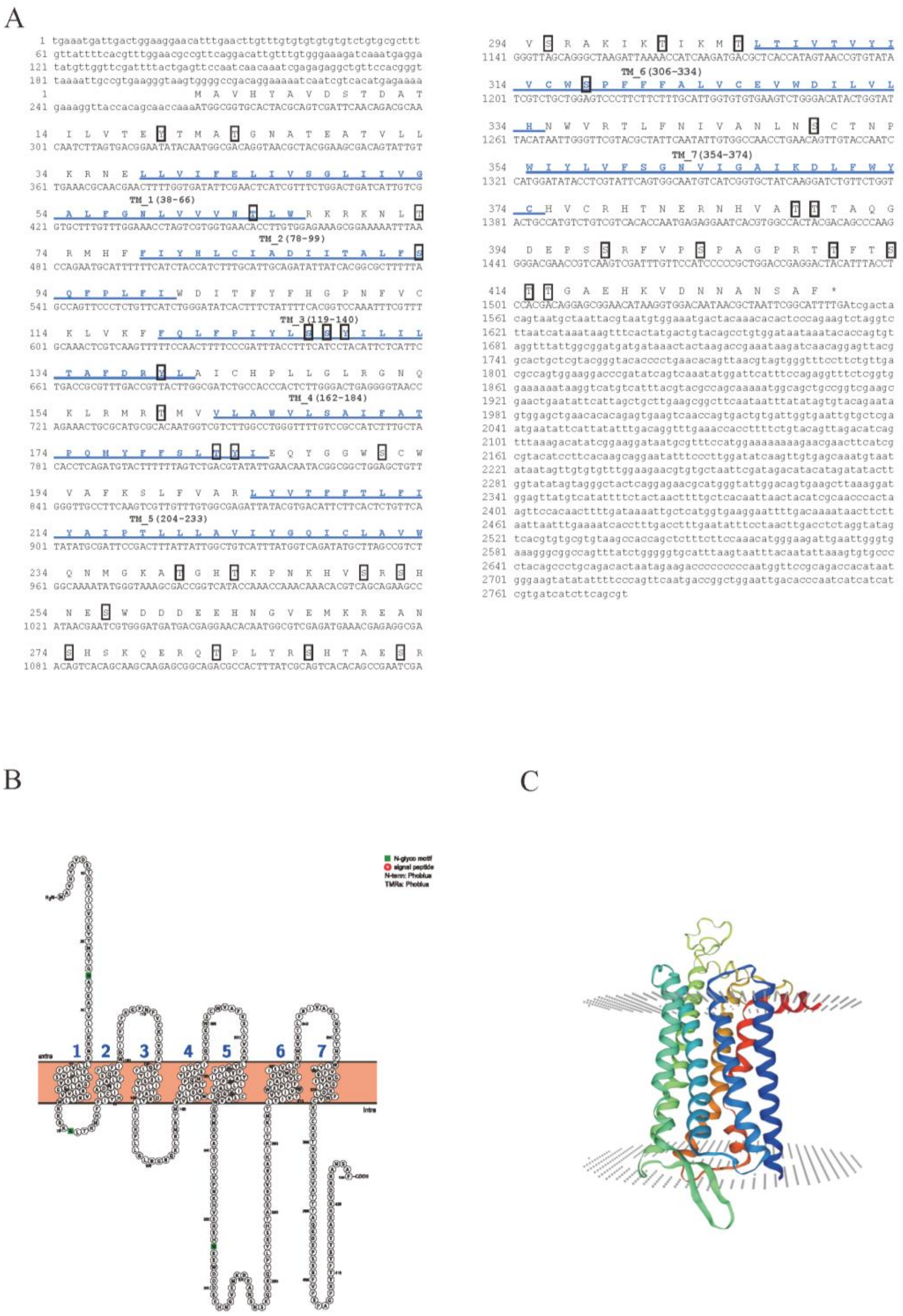

Figure S2. Molecular structure and characterization of *AjHOR* in *A. japonicus*. (**A**) *AjHOR* cDNA sequence (GenBank accession number: PIK60987.1) and the deduced amino acid sequence. ORF were denoted in uppercase; 3'UTR and 5'UTR were shown in lowercase. The asterisk (*) indicated the termination codon. The square indicated phosphorylation sites. The seven transmembrane domains (TM1-TM7) were labeled in blue underline. (**B**) Seven transmembrane domains of *AjHOR*. The number showed the position of seven transmembrane domains. (**C**) Protein tertiary structure of *AjHOR*.

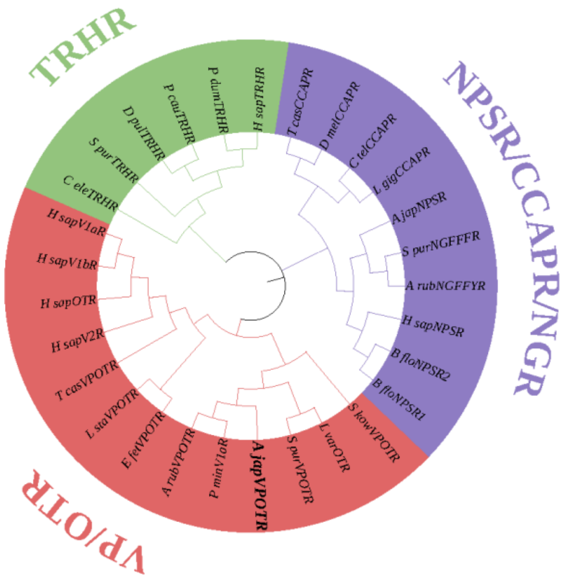

Figure S3. Phylogenetic tree based on amino acid sequences of VP/OT-type receptors. The phylogenetic relationship of VP/OT-type, NPS/CAP/NG-type and TRH-type neuropeptide receptors from different species. The Genbank numbers were listed in Supplementary Table S3.

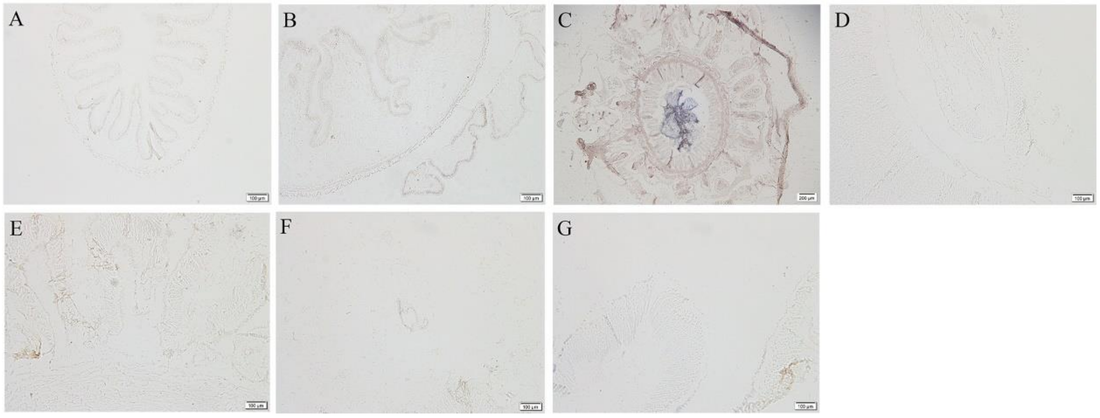

Figure S4. Tissues incubated with sense probe as the negative controls in adult *A. japonicus*. (**A**) Intestine; (**B**) Mesentery; (**C**) Oral position: water vascular system, tentacle and circumoral nerve ring; (**D**) Longitudinal muscle; (**E**) Body wall; (**F**) Water canal; (**G**) Tube foot.

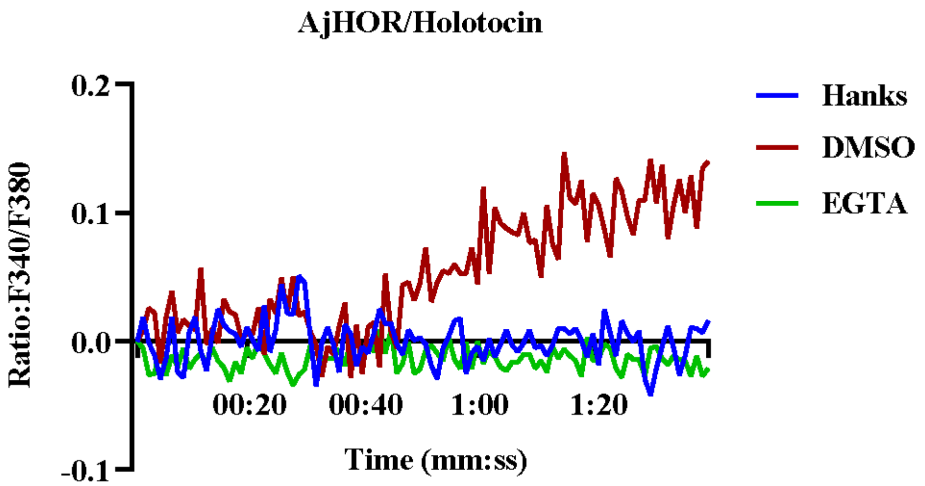

Figure S5. Calcium chelators EGTA (5 mM) on holotocin (1 μM) in DMSO-mediated Ca^{2+} influx in HEK293T cells. All experiments were repeated independently three times with similar results.

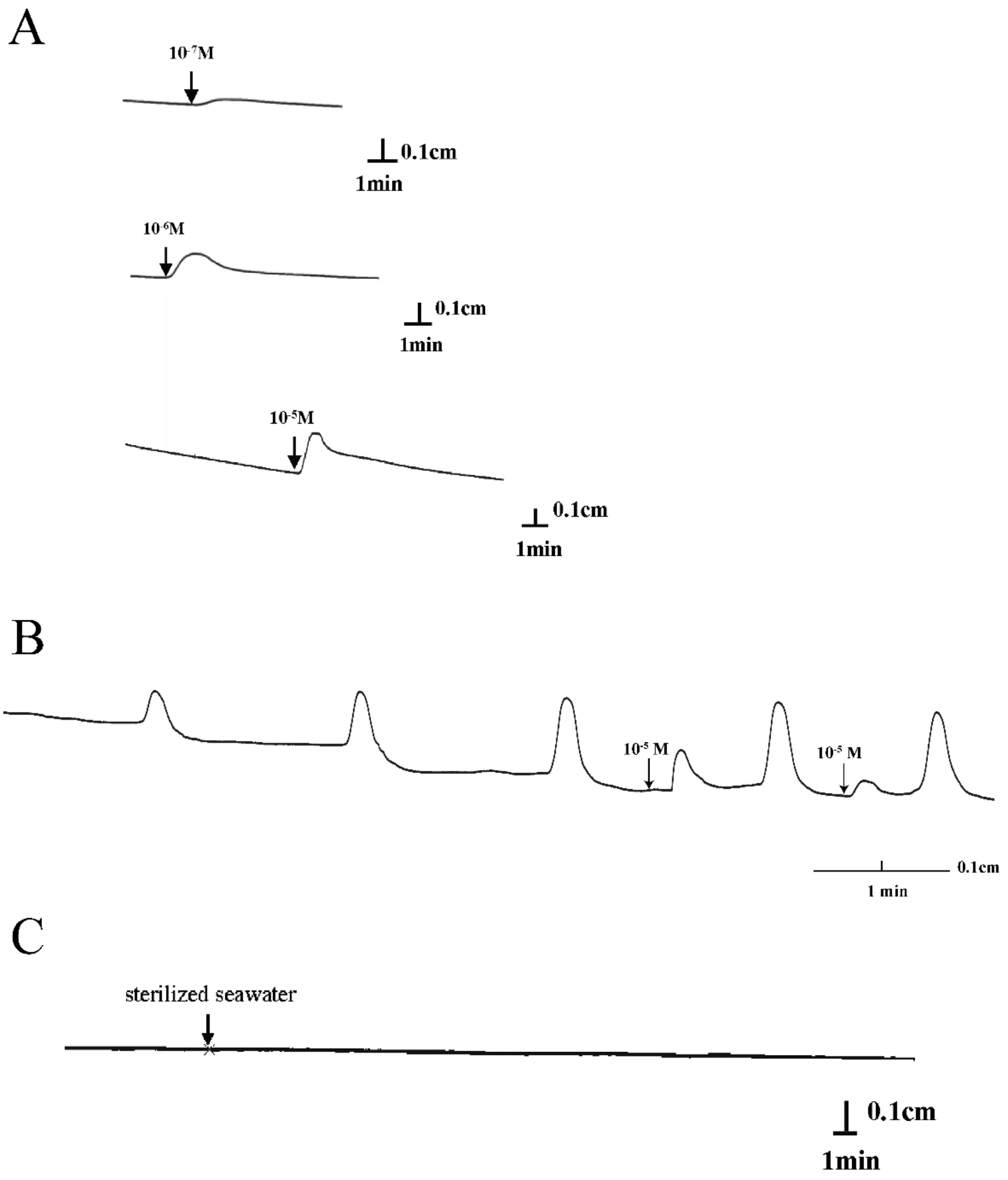

Figure S6. Negative and positive controls of *in vitro* pharmacological experiments and the necessity detection of calcium ions during longitudinal muscle contraction caused by holotocin. (**A**) NGIWYamide caused tonic contraction on longitudinal muscle preparations from *A. japonicus*. (n = 3). (**B**) A representative recording showed that holotocin (10^{-5} M) caused contraction of tentacle preparation. (**C**) Sterile seawater was used as the negative control and it could not cause the contraction of preparations.

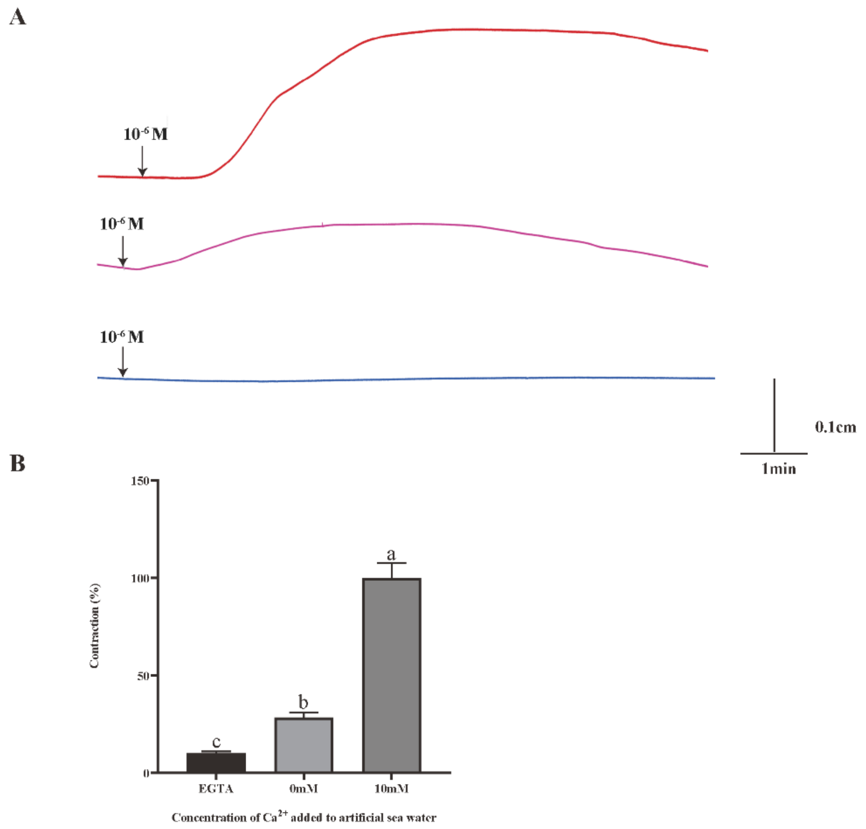

Figure S7. Dependence of holotocin contraction on different calcium ion concentration (Ca^{2+}-free solution containing 5 mM EGTA, 0 mM, 10 mM). (**A**) A representative recording showed that holotocin (10^{-6} M) caused longitudinal muscle contraction on artificial sea water with 0 mM or10 mM Ca^{2+}. EGTA was used to chelate calcium ions and holotocin could not cause longitudinal muscle contraction. (**B**) Comparison of contraction efficiency of holotocin with different Ca^{2+} concentration, the contraction efficiency of holotocin in 10 mM Ca^{2+} was defined as 100%. The significant difference ($p < 0.05$) was shown by lowercase letters. Values were mean ± S.E.M (n = 3 biological replicates).

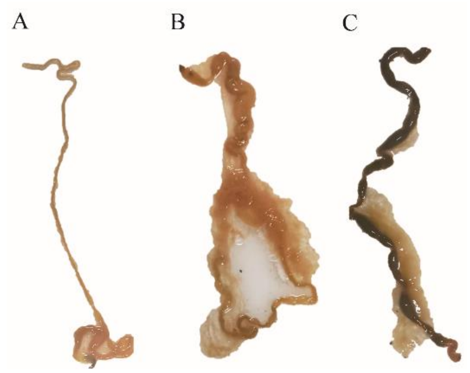

Figure S8. The anatomy of intestine during long-term injection in different groups. (**A**) The state of intestine in HOL. (**B**) The state of intestine in HOH. (**C**) The state of intestine after injection in CO.
