# Supplementary material for: A Putative Role of Vasopressin/Oxytocin-Type Neuropeptide in Osmoregulation and Feeding Inhibition of Apostichopus japonicus
Source: Int J Mol Sci. 2023 Sep 20;24(18):14358. doi: 10.3390/ijms241814358 (PMC10532012; doi:10.3390/ijms241814358)
Supplement: Supplementary file 1 [file ijms-24-14358-s001.zip › Supplementary Tables.pdf]

Table S1. Accession numbers or citations of VP/OT-type peptide and precursor in deuterostomes.

Classification	Abbreviations	Full Species name	Accession numbers or citations
Deuterostomes	*H_sapVP*	*Homo sapiens (vasopressin)*	M25647
	H_sapOT	*Homo sapiens (oxytocin)*	M25650
	S_pli	*Styela plicata*	AB255884
	C_int	*Ciona intestinalis*	AB432887
	B_flo	*Branchiostoma floridae*	XM_002608950
	S_kow	*Saccoglossus kowalevskii*	FF512954
	S_pur	*Strongylocentrotus purpuratus*	XM_003724442.2
	A_jap	*Apostichopus japonicus*	MF401997
	H_gla	*Holothuria glaberrima*	[53]
	H_sca	*Holothuria scabra*	
	A_rub	*Asterias rubens*	GenBank: KT601711
	A_pla	*Acanthaster planci*	[36]
	O_vic	*Ophionotus victoriae*	[48]
	O_ara	*Ophiopsila aranea*	
	A_fil	*Amphiura filiformis*	
	An_jap	*Anneissia japonica*	[47]
	A_med	*Antedon mediterranea*	
	F_ser	*Florometra serratissima*	

Table S2. Primers used for RACE, RT-qPCR analysis and Plasmid construction.

Primers	Sequence (5'-3')	Used
Holotocin precursor-R	GATTACGCCAAGCTTTGGCTAGGCTT TCAGTGTGTGGAGT	5'RACE
AjHOR-R	GATTACGCCAAGCTTAAATGTAGTCC TCGGTCCAGCGGGG	5'RACE
AjHOR-F	GATTACGCCAAGCTTGAATCACGTGG CCACTACGACAGCC	3'RACE
Holotocin precursor-F	ATGTGTTCTGTGCCGCTCTT	RT-qPCR
Holotocin precursor-R	GCAATGGTCTCCTACGACTTCA	
AjHOR-F	ATGATGACGAGGAACACAATGG	
AjHOR-R	AGCAGACGATATACACGGTTACT	
AjCCKP1-F	GTTAGTCAACCCGAAGGA	
AjCCKP1-R	TGAATATCGTGCGAGGAG	
AjCCKP2-F	TCAGTAATATGCGTCCTTGCT	
AjCCKP2-R	TCCAGTGCTTCCAGTTCATC	

β-Actin-F	AAGGTTATGCTCTTCCTCACGCT	
β-Actin-R	GATGTCACGGACGATTTCACG	
β-Tubulin-F	GAAAGCCTTACGACGGAACA	
β-Tubulin-R	CACCACGTGGACTCAAAATG	
Holotocin precursor-F(ISH)	CCACTGTGAGGTGAATGTGCAAGATGTC	*In situ* hybridization
Holotocin precursor-R(ISH)	TGTCTCCAATACCGCCTGGCTCGTA	
AjHOR-F(PC)	CGCGGATCCAAAATGGCGGTGCACTACGC	pcDNA3.1(+) plasmid constructed
AjHOR-R(PC)	CCGGAATTCTCAAAATGCCGAATTAGCGT	
AjHOR-F(EGFP)	CCGGAATTCTGAAAATGGCGGTGCACTACGC	EGFP plasmid constructed
AjHOR-R(EGFP)	CGCGGATCCCGAAATGCCGAATTAGCGTTAT	

Table S3. Accession numbers of VP/OT-type, NPS/CCAP/NG peptide-type, and TRH-type neuropeptide receptors.

Abbreviations	Full species name	Accession numbers
H_sapV1aR	*Homo sapiens*	NM_000706.4
H_sapV1bR	*Homo sapiens*	NM_000707.4
H_sapV2R	*Homo sapiens*	NM_000054.5
H_sapOTR	*Homo sapiens*	NM_001354655.1
A_japVPOTR	*Apostichopus japonicus*	PIK60987
S_kowVPOTR	*Saccoglossus kowalevskii*	XP_002735546
S_purVPOTR	*Strongylocentrotus purpuratus*	XM_779280.4
A_rubVPOTR	*Asterias rubens*	MK279533
L_staVPOTR	*Lymnaea stagnalis*	U27464.1 [a]
E_fetVPOTR	*Eisenia fetida*	AB121771.1
T_casVPOTR	*Tribolium casteneum*	EU128495.1
H_sapNPSR	*Homo sapiens*	AY310326.1
B_floNPSR1	*Branchiostoma floridae*	XP_002605431
B_floNPSR2	*Branchiostoma floridae*	XP_002605430
A_japNPSR	*Apostichopus japonicus*	PIK35938
A_rubNGFFYR	*Asterias rubens*	KP171535
S_purNGFFFR	*Strongylocentrotus purpuratus*	KP171538
L_gigCCAPR	*Lottia gigantea*	XP_009061331
C_telCCAPR	*Capitella teleta*	ELU12393
D_melCCAPR	*Drosophila melanogaster*	AY219842
T_casCCAPR	*Tribolium casteneum*	NM_001083326
H_sapTRHR	*Homo sapiens*	ENSG00000174417
S_purTRHR	*Strongylocentrotus purpuratus*	XP_783972.2
P_dumTRHR	*Platynereis dumerilii*	AKQ63029
P_cauTRHR	*Priapulus caudatus*	XP_014663378
D_pulTRHR	*Daphnia pulex*	ADZ15312
C_eleTRHR	*Caenorhabditis elegans*	CCD66234
L_varOTR	*Lytechinus variegatus*	XM_041620700.1
P_minV1aR	*Patiria miniata*	XM_038193257.1
